# Crimean-Congo Hemorrhagic Fever Virus Africa 1 Lineage in *Hyalomma dromedarii* Ticks, Algeria, 2023

**DOI:** 10.3201/eid3108.250123

**Published:** 2025-08

**Authors:** Marbouha Temani, Aissam Hachid, Rafik Garni, Amir Abderezzak Guessoum, Mohammed Hocine Benaissa, Ahmed Fayez Khardine, Abdelhakim Kimouche, Ahcéne Hakem, Idir Bitam, Kamal Eddine Benallal, Ismail Lafri

**Affiliations:** Institut Pasteur d’Algérie, Algiers, Algeria (M. Temani, A. Hachid, R. Garni, A.A. Guessoum, A.F. Khardine, K.E. Benallal, I. Lafri); Université Alger 1 Faculté de Pharmacie, Algiers (A. Hachid); Centre de Recherche Scientifique Et Technique Sur Les Régions Arides, Touggourt, Algeria (M.H. Benaissa); Inspéction Vétérinaire, Direction des Services Agricoles de la Wilaya d’Illizi, Illizi, Algeria (A. Kimouche); Centre de Recherche en Agropastoralisme, Djelfa, Algeria (A. Hakem, I. Bitam); Université de Blida 1, Blida, Algeria (I. Lafri)

**Keywords:** Crimean-Congo hemorrhagic fever, Crimean-Congo hemorrhagic fever virus, viruses, ticks, vector-borne infections, Hyalomma dromedarii, camels, dromedaries, Algeria

## Abstract

We conducted a Crimean-Congo hemorrhagic fever virus (CCHFV) survey of *Hyalomma* spp. ticks collected from camels in southeastern Algeria. Of 138 tick pools, 1 was CCHFV positive; the sequenced strain belonged to the Africa 1 genotype. Healthcare professionals in Algeria should be aware of this detection of a circulating pathogenic CCHFV genotype.

Infection with Crimean Congo hemorrhagic fever virus (CCHFV; *Orthonairovirus hemorrhagiae*; Nairoviridae: Bunyavirale) provokes fever and hemorrhagic manifestations in humans but results in asymptomatic infections in animals (*1*). CCHFV is maintained in nature through wild and domestic animals serving as amplification hosts and ticks as reservoirs. CCHFV is endemic to Africa, the Middle East, Asia, and Europe (*2*). However, knowledge of CCHFV in North Africa is limited to few serologic surveys and molecular characterization in ticks. 

In Algeria, Agai virus (*Orthonairovirus parahemorrhagiae*), previously known as AP92-like CCHFV, has been detected in *Hyalomma aegyptium* ticks collected from tortoises (*3*). In addition, 2 seroprevalence studies of CCHFV conducted on dromedary camels (*Camelus dromedarius*) in different regions from southern Algeria showed a high rate of IgG against CCHFV (*2*,*4*). We aimed to detect CCHFV among ticks in southern Algeria, where serologic evidence of the virus was reported among camels.

During September–November 2023, we conducted surveillance for CCHFV in ticks collected from camels in the Wilayates (provinces) of Ouargla, Illizi, and Djanet, located in southeastern Algeria (Appendix). We morphologically identified ticks by using taxonomic keys and pooled specimens on the basis of species, sex, developmental stage, feeding status, and collection sites; we stored pools at −80°C until analysis. We cleaned ticks with 70% ethanol and then crushed them by using a Retsch MM 400 Mixer Mills (https://www.retsch.com). We extracted nucleic acid material (RNA and DNA) from supernatants by using NucleoSpin Virus kits (Macherey-Nagel, https://www.mn-net.com), according to the manufacturer’s instructions. We screened tick extracts for CCHFV by using real-time reverse transcription PCR (RT-PCR) targeting the small (S) segment of CCHFV (*5*) and confirmed positive pools by using an endpoint RT-PCR targeting the S segment of the *Nairovirus* group (*6*), followed by Sanger sequencing. We molecularly confirmed positive pools by sequencing the mitochondrial cytochrome oxidase I gene (*7*). We constructed a maximum-likelihood tree with 1,000 bootstrap replicates using a Tamura 1992 with gamma distribution substitution model (*8*) using different CCHFV sequence genotypes (Figure). We deposited the sequence obtained in this study into GenBank (accession no. PQ246052).

**Figure:**
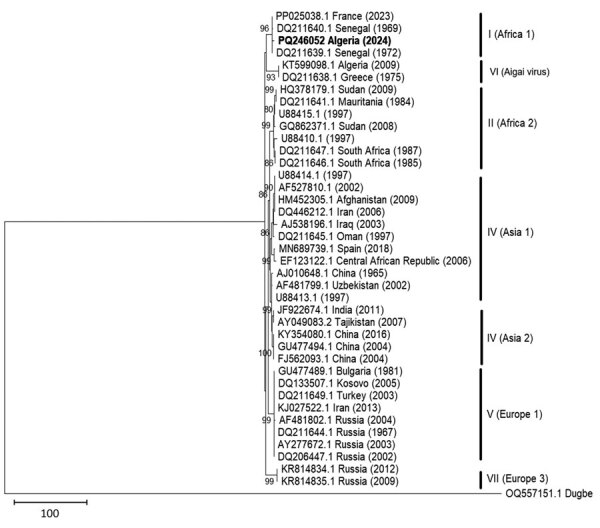
Phylogenetic analysis of the small segment sequence of Crimean-Congo hemorrhagic fever virus Africa 1 lineage detected in ticks collected from camels in southeastern Algeria, 2023. Bold indicates the strain detected in Algeria; other sequences are labeled by GenBank accession number, geographic origin, and sampling year. Only bootstrap values >80 are shown. Scale bar indicates substitutions per site.

We grouped a total of 346 ticks collected from 103 camels into 138 pools. Tick species consisted of 290 (83.81%) *Hy. dromedarii*, 26 (7.51%) *Hy. rufipes*, 19 (5.49%) *Hy. impeltatum*, and 11 (3.17%) *Hy. impressum* (Table). Each pool contained 1–5 ticks grouped by feeding status, species, locality, and sex. Three pools tested positive for CCHFV by the first real-time RT-PCR: pool 22 (cycle threshold [Ct] value = 26.91), pool 19 (Ct = 36.24), and pool 13 (Ct = 39.04). Only pool 22, containing 5 male *Hy. dromedarii* ticks, generated a 465-bp fragment of the S segment using the endpoint RT-PCR. A maximum-likelihood tree showed that the Algeria sequence formed a monophyletic group or cluster with strains from Senegal and France belonging to the Africa 1 genotype (GenBank accession nos. DQ211639, DQ211639, and PP025038) with 95% bootstrap support (Figure). Molecular identification of ticks in positive pools using cytochrome oxidase I gene confirmed the presence of *Hy. dromedarii* and *Hy. impeltatum* ticks (Table), both species are known as competent vectors for CCHFV.

**Table:** Description of CCHFV in tick pools collected from camels in southeastern Algeria, 2023*

Wilayates (province)	Tick species	No. pools	No. ticks/pool, by sex	Positive pool no./Ct/sex
Ouargla	*Hyalomma dromedarii*	45	20 M, 25 F	
	*Hy. rufipes*	2	1 M, 1 F	
Illizi	*Hy. dromedarii*	60	23 M, 37 F	13/39.04/M; **22/26.91/M**
	*Hy. rufipes*	4	1 M, 3 F	
	*Hy. impeltatum*	6	1 M, 5 F	19/36.24/M
	*Hy. impressum*	2	2 F	
Djanet	*Hy. dromedarii*	13	4 M, 9 F	
	*Hy. impeltatum*	4	4 F	
	*Hy. impressum*	2	2 M	

*Pools of ticks were tested with quantitative reverse transcription PCR for the small segments of CCHFV RNA. A total of 138 pools, comprising 346 ticks collected from 103 camels, were tested for CCHFV. Only 3 pools of *Hy. dromedarii* ticks from Illizi were CCHFV positive; only the pool in bold, containing 5 male *Hy. dromedarii* ticks, generated a 465-bp fragment of the small segment using the endpoint RT-PCR. CCHFV, Crimean-Congo hemorrhagic fever virus; Ct, cycle threshold.

We detected and characterized a pathogenic strain of CCHFV in local tick populations collected from camels in southern Algeria, underscoring circulation of the virus in this region. Camels play a vital economic and cultural role in the region, especially through transhumance. However, movements between Algeria and endemic areas in neighboring countries through legal and illegal cross-border trade increase the likelihood of encountering viremic animals and tick vectors. Moreover, migratory birds from the Trans-Saharan Flyway carrying *Hyalomma* spp. ticks are likely a major source of CCHFV strains circulating between Africa and Europe, as reported in Morocco and France (*9*,*10*). Our findings suggest that the possible pathway of CCHFV dissemination to Algeria from endemic areas could involve migratory birds, considering that the CCHFV Africa 1 strain identified in this study is phylogenetically closely related to the strains previously reported in Corsica (France) and Senegal. The potential for the continuous spread of CCHFV across Algeria and North Africa is substantial. Indeed, Algeria’s large territory harbors various tick species known for their CCHFV transmission competence, increasing the likelihood of CCHFV circulation among ticks and animals. This study, limited to 3 provinces in the Sahara, serves as a starting point for broader epidemiologic studies across the country; expanding surveillance to other regions, animals, humans, and tick vectors is crucial for informing policy-makers and enabling a comprehensive risk assessment of CCHFV exposure in Algeria. Using next-generation sequencing technologies for whole-genome sequencing of CCHFV will enable detailed genomic characterization and clarify spatiotemporal transmission dynamics. 

In summary, our results document detection of a CCHFV pathogenic genotype among camels in Algeria, carried by *Hyalomma* spp. ticks. Healthcare professionals should be aware of CCHFV circulation in this region and the resulting potential for human infection.

Appendix Additional information for Crimean-Congo hemorrhagic fever virus Africa 1 lineage in *Hyalomma dromedarii* ticks, Algeria, 2023.
