# Supplementary material for: Crimean-Congo Hemorrhagic Fever Virus Africa 1 Lineage in Hyalomma dromedarii Ticks, Algeria, 2023
Source: Emerg Infect Dis. 2025 Aug;31(8):1673–6. doi: 10.3201/eid3108.250123 (PMC12309754; doi:10.3201/eid3108.250123)
Supplement: Appendix — Additional information for Crimean-Congo hemorrhagic fever virus Africa 1 lineage in Hyalomma dromedarii ticks, Algeria, 2023. [file 25-0123-Techapp-s1.pdf]

Article DOI: https://doi.org/10.3201/eid3108.250123

EID cannot ensure accessibility for supplementary materials supplied by authors. Readers who have difficulty accessing supplementary content should contact the authors for assistance.

Crimean-Congo Hemorrhagic Fever Virus Africa 1 Lineage in *Hyalomma dromedarii* Ticks, Algeria, 2023

Appendix

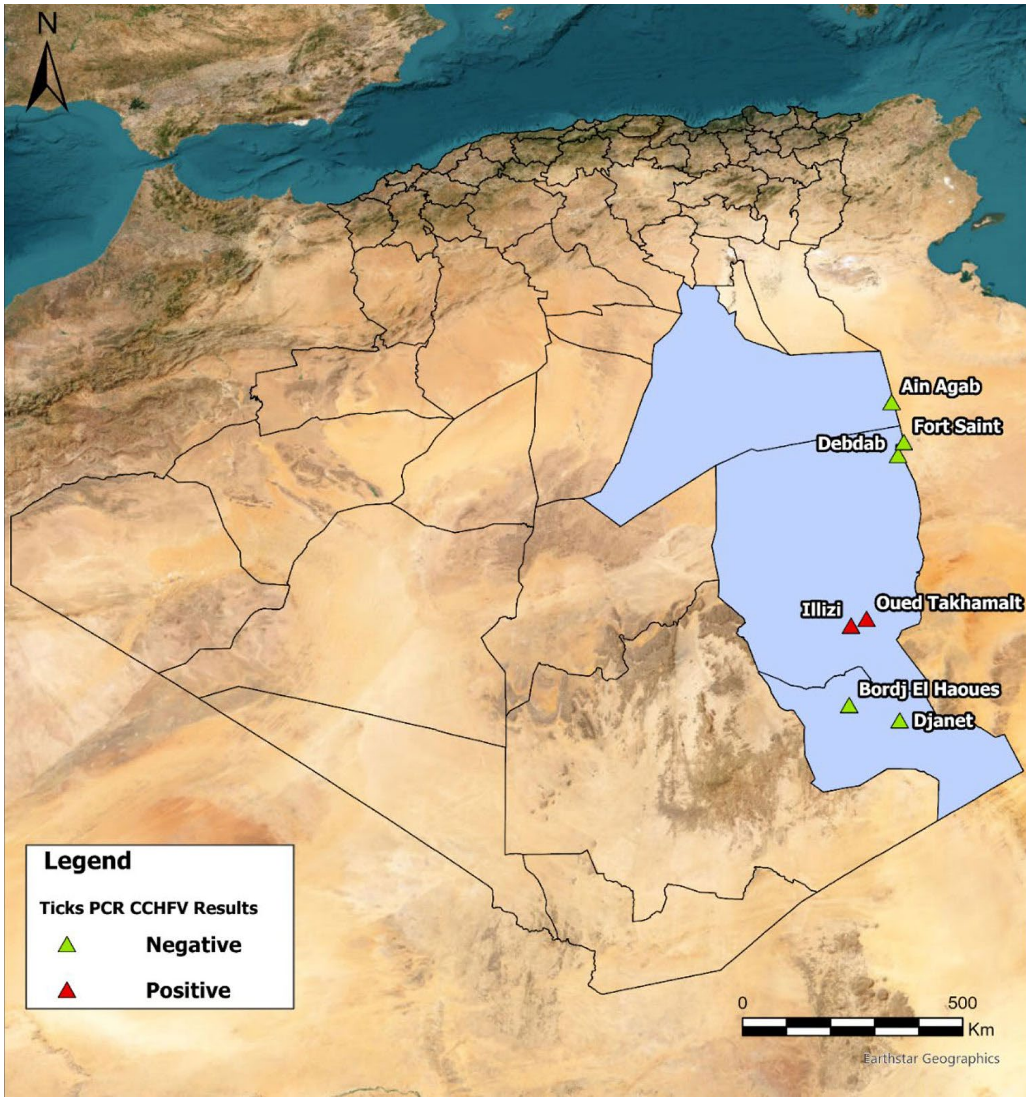

Appendix Figure. Map showing study area and tick sampling locations for study of Crimean-Congo hemorrhagic fever virus detected in ticks collected from camels in southeastern Algeria, 2023.
